# Supplementary material for: Vitamin B5 (d-pantothenic acid) localizes in myelinated structures of the rat brain: Potential role for cerebral vitamin B5 stores in local myelin homeostasis
Source: Biochem Biophys Res Commun. 2020 Jan 29;522(1):220–5. doi: 10.1016/j.bbrc.2019.11.052 (PMC6977085; doi:10.1016/j.bbrc.2019.11.052)
Supplement: Multimedia component 1 [file mmc1.docx]

**Ismail et al**

**Suppl. Material**

**Suppl. Methods**

*Animals*

Adult male Wistar rats (220-250 g; N = 12) were randomly assigned to one of two treatment groups and injected intraperitoneally after an overnight fast with either 55 mg/kg STZ in 0.9% w/v NaCl solution (N = 6) or an equivalent volume of saline only (N = 6). Hyperglycemia was confirmed 3 days after STZ injection. Animals were housed in groups of two or three under a 12-h light-dark cycle, at 22 ± 2 ^o^C and 60% humidity, with ad libitum access to food and water. After 16 weeks, rats were terminally anesthetized (isoflurane 2% v/v in O_2_) [1], and whole brains removed and frozen at -80°C until further dissected. Subsequently, brains were divided sagittally into two hemispheres, one being used for histological/immunohistochemical (IHC) studies, and the other for metabolomics and metallomics.

*Tissue Processing for Microscopy*

Tissue was fixed (≥ 16 h; 4% w/v paraformaldehyde in phosphate-buffered saline (PBS) pH 7.4; Fisher Chemical, UK); placed overnight in a sucrose solution (10% w/v); then transferred (≥ 16 h) to sucrose (30% w/v). Sections were then embedded (KP-Cryo-Compound Frozen Tissue Medium; Klinipath B. V., Duiven, Netherlands) and, once set, stored at -80°C until final sectioning. Serial sagittal sections (6-μm/30 sections per brain; CM3050S cryostat; Leica, Wetzlar, Germany) were cut, then mounted (X-tra adhesive microslides; Leica) for histological or IHC staining.

*Histological staining*

Sections were stained with H&E (haematoxylin and eosin; ST5010 Autostainer XL, Leica) or Nissl stain (Luxol Fast Blue (LFB)/Cresyl Violet). For Nissl staining, slides were kept at ~56°C overnight (not > 16 h) in LFB solution (0.1 % w/v). After rinsing with distilled water, lithium carbonate solution (0.05% w/v) was applied for 30 s, followed by ethyl alcohol (70% v/v) for ~30 s for differentiation (until gray matter was pale), then slides were again rinsed in distilled water and examined microscopically to confirm suitable gray/white matter differentiation. Nissl staining identifies myelin containing structures and neuronal nuclei. To stain the neuropil, sections were counterstained with cresyl violet (0.1% w/v) for 30 s, and then further differentiated by washing with ethyl alcohol (95% v/v) then 100% twice for 5 min, followed by 2x5-min washes in xylene. Coverslips were then added to the slides (Automated Glass Coverslipper CV5030; Leica). Finally, images were prepared (Panoramic 250 Slide Scanner; 3DHISTECH, Budapest, Hungary) and visualized (CaseViewer software; 3DHistech, Germany).

*Immunohistochemistry*

Slides were briefly washed with acetone and then PBS, and then incubated for 1 h with a blocking buffer (bovine serum albumin (1.5 mg; A3059, Sigma, USA), 15 μl normal goat serum, and 1.5 μl Triton X-100 (X100; Sigma) diluted to 1500 μl final volume with PBS). The primary antibody used was anti-conjugated D-pantothenate antibody (AP090; GemacBio, Saint Jean d’Illac, France) [2] at the optimised dilution of 1:500 in PBS, and multiple sections per slide were separated with an ImmEdge hydrophobic barrier pen, (H-4000; Vector, Burlington, USA). Slides were incubated with the primary antibody solution at 40°C overnight, and then washed thoroughly with PBS. They were then incubated for 60 min at room temperature with Alexafluor 568 (1:1000; Fisher Chemical, UK) diluted in PBS. Following 3 further washes with PBS, DAPI (1:1000 in PBS), (2-[4-amidinophenyl-1H-indole-6-carboxamidine]), was applied for 5 min at room temperature. Slides were mounted with coverslips (Vectashield HardSet mounting medium, H-1440; Vector; Burlington, VT, USA). One section from each sample served as a negative immunofluorescence control with omission of the primary antibody and addition of an equivalent volume of PBS only. Images were obtained (Panoramic 250 Slide Scanner; 3DHistech) using the DAPI, FITC and Texas Red channels, and viewed (CaseViewer; 3DHistech).

*Gas chromatography mass spectrometry (GC-MS)*

50±5 mg samples of tissue from each brain were transferred to 2-ml Safe-Lock tubes (Eppendorf, Germany) and frozen at -80°C prior to tissue extraction. A Folch-style extraction procedure was used. A mixture of internal labelled standards was used: the first contained succinic acid d4, and glycine d5 in 10 ml 50:50 (v/v) methanol:water; the second citric acid d4, fructose 13C6, and tryptophan d5, also in 10 ml 50:50 (v/v) methanol:water; the third leucine d10, alanine d7 in 10 ml 50:50 (v/v) methanol:water; and the fourth stearic acid d35 and benzoic acid d5 in 10 ml methanol. 10 mg of each reagent was used. 1 ml from each internal standard was added to 6 ml of methanol and then further diluted to a final volume where the internal standard mixture in methanol was 1:16.

800 μl of the 50:50 (v/v) chloroform:methanol mixture containing the internal standards was added to each sample. A 3-mm Tungsten carbide bead was added to each tube, and the samples were extracted using the TissueLyser (Qiagen; UK), at a frequency of 25 Hz for 10 min. 400 μl of water was added to each sample; they were then vortexed and placed in a centrifuge for 15 min at 2400 x g. 200 μl was collected from the methanol phase of each sample and transferred to individual 2-ml microcentrifuge tubes. An additional 200 μl of each sample, excluding the extraction blank, contributed to a pool, from which 200-μl quality control (QC) samples were derived. The samples were dried overnight and then stored at 4°C.

Metabolites were derivatised by re-dissolution in 60 μl of methoxylamine HCl (226904, Sigma) solution (20 mg/ml in dry pyridine) and heated at 80°C for 20 min. Then, 60 μl of MSTFA (*N*-methyl-*N*-(trimethylsilyl)-trifluoroacetamide; 223298, Sigma) was added, and samples were re-incubated at 80°C for 20 min. Finally, 10 μl of a retention-time marker solution (nine n-alkanes covering the range C12-C32 dissolved at 10 mg/ml in 1:1 hexane:pyridine) was added, the samples were centrifuged at 15,800 g for 15 min, and 90 μL of the resulting supernatant from each extract was transferred to autosampler vials for GC/MS analysis.

*ICP-MS*

Digestion acid containing a 1.5 ml Internal Standard Mixture (5183-4681; Agilent Technologies; Cheadle, UK) and 30 ml concentrated nitric acid (A509 Trace Metal Grade; Fisher, Loughborough, UK) was prepared in metal-free centrifuge tubes (525-0631; VWR, Lutterworth, UK), and 200 μl of the digestion acid was added to each sample. Sample-containing tubes were placed on a Dri-Block heater at ambient temperature, after which the temperature was raised to 60^o^C for 30 min, and raised thereafter to 100^o^C for a further 3.5 h. Following digestion, tubes were removed from the heater and left to cool for 30 min. 100 μl of each digest was diluted with 5 ml MilliQ water (Merck KGaA; Darmstadt, Germany) in a 15-ml metal-free centrifuge tube. A 2% (v/v) dilute acid solution was prepared, with 20 ml of the previously prepared digestion acid added to 980 ml MilliQ water in a polypropylene volumetric flask. Calibration standards contained 2% (v/v) nitric acid solution, and Environmental Calibration Standard Mixture (5183-4688; Agilent Technologies, Cheadle, UK); two digestion blanks were also produced.

Data were captured in Microsoft Excel worksheets. Results for each sample were corrected to wet-weight and then for the dilution of acid used. Elements measured were Na, K, Mg, Ca, Mn, Fe, Cu, Zn, and Se. Mean ±95% confidence intervals were compared using Prism v7.04 (GraphPad; La Jolla, CA). Significance was determined as p < 0.05.

**Suppl. Discussion**

Urea content of the diabetic rat brain was significantly increased, consistent with the severe increases reported across several brain regions in HD, including those in cases with pre-symptomatic disease [3-5]. Increased urea levels exert toxicity through carbamoylation of free amino groups in proteins [6]. The marked increases of brain urea in HD and diabetes are significant, as they could indicate that defective cerebral urea metabolism may contribute to impaired neuronal function in both diseases.

The TCA cycle is essential for cerebral energy utilization [7]. Here, levels of the TCA-cycle intermediates 2-oxoglutaric acid and succinic acid were elevated in the diabetic brain. Similar abnormalities in TCA-cycle intermediates are present in HD brain [4, 8], so the TCA cycle provides a further point of confluence between these two states.

In the brain, certain amino acids can act as precursors for the synthesis of neurotransmitters, and as substrates for gluconeogenesis. Here we found increased levels of isoleucine and leucine in the diabetic brain: these branched-chain amino acids serve as substrates for cerebral gluconeogenesis [9], a key pathway of endogenous glucose production activated during starvation and other causes of impaired glucose utilization. Gluconeogenesis is frequently upregulated in diabetes [10], and our current findings are consistent with elevated cerebral gluconeogenesis. In HD brain, there are typically perturbations in the amino acids which are precursors for neurotransmitters, such as phenylalanine (dopamine, adrenaline and noradrenaline) and glutamine (glutamate) [4]. However, the trends in amino acid levels that we found here in diabetic brain differed from those in HD. For example, whereas phenylalanine is increased in several HD-brain regions [4], our current study found a significant decrease in diabetic brain. Therefore, there are important differences between amino-acid perturbations in HD as compared with diabetic brain.

**Suppl. References**

[1] O.J. Freeman, R.D. Unwin, A.W. Dowsey, et al, Metabolic dysfunction is restricted to the sciatic nerve in experimental diabetic neuropathy, Diabetes, 65 (2016) 228-238.

[2] A. Mangas, J. Yajeya, N. Gonzalez, et al, Detection of pantothenic acid-immunoreactive neurons in the rat lateral septal nucleus by a newly developed antibody, Folia Histochem Cytobiol, 54 (2016) 186–192.

[3] S. Patassini, P. Begley, S.J. Reid, et al, Identification of elevated urea as a severe, ubiquitous metabolic defect in the brain of patients with Huntington's disease, Biochem Biophys Res Commun, 468 (2015) 161-166.

[4] S. Patassini, P. Begley, J. Xu, et al, Metabolite mapping reveals severe widespread perturbation of multiple metabolic processes in Huntington's disease human brain, Biochim Biophys Acta, 1862 (2016) 1650-1662.

[5] R.R. Handley, S.J. Reid, R. Brauning, et al, Brain urea increase is an early Huntington’s disease pathogenic event observed in a prodromal transgenic sheep model and HD cases, Proc Natl Acad Sci USA, 114 (2017) E11293-E11302.

[6] M. D’Apolito, X. Du, H. Zong, et al, Urea-induced ROS generation causes insulin resistance in mice with chronic renal failure, J Clin Invest, 120 (2010) 203–213.

[7] H.A. Krebs, The history of the tricarboxylic acid cycle, Perspect Biol Med, 14 (1970) 154–172.

[8] N.N. Naseri, J. Bonica, H. Xu, et al, Novel metabolic abnormalities in the tricarboxylic acid cycle in peripheral cells from Huntington's disease patients, PloS ONE, 11 (2016) e0160384.

[9] P. Felig, Amino acid metabolism in man, Annu Rev Biochem 44 (1975):933–955.

[10] R. Basu, V. Chandramouli, B. Dicke, B. Landau, R.A. Rizza, Obesity and type 2 diabetes impair insulin-induced suppression of glycogenolysis as well as gluconeogenesis, Diabetes, 54 (2005) 1942–1948.

| Suppl. Table 1  Levels of all measured metabolites in whole-brain tissue from control and diabetic adult-male Wistar rats. Fold-changes are diabetic/control. Changes with P < 0.05 (10% FDR) are considered significant and shown in bold-italic font. | | | |
| --- | --- | --- | --- |
| **Metabolite** | **Control**  **Mean (95% CI)** | **Diabetic**  **Mean (95% CI)** | **Fold-Change** |
| 2-Hydroxyglutaric acid (D) | 0.063 (0.028-0.099) | 0.043 (0.035-0.051) | 0.7 |
| 2-Oxoglutaric acid (C) | 0.12 (0.088-0.15) | 0.22 (0.17-0.28) | ***1.9*** |
| 2-Pyrrolidone-5-carboxylic acid (D) | 0.31 (0.25-0.36) | 0.28 (0.18-0.37) | 0.9 |
| Adenine (D) | 0.19 (0.14-0.24) | 0.22 (0.16-0.27) | 1.2 |
| Adenosine (D) | 1.4 (0.055-2.2) | 1.8 (1.03-2.5) | 1.3 |
| Alanine (D) | 0.021 (0.016-0.027) | 0.030 (0.020-0.040) | 1.4 |
| Benzoic acid (C) | 0.018 (0.014-0.022) | 0.020 (0.012-0.027) | 1.1 |
| beta-Alanine (D) | 0.11 (0.074-0.14) | 0.11 (0.10-0.12) | 1.0 |
| ***beta-Hydroxybutyric acid (D)*** | ***0.0024 (0.0017-0.0031)*** | ***0.0086 (0.0041-0.013)*** | ***3.6*** |
| Cholesterol (C) | 0.043 (-0.0074 to 0.094) | 0.090 (-0.0011 to 0.18) | 2.1 |
| Citric acid (C) | 0.36 (0.32-0.40) | 0.32 (0.26-0.38) | 0.9 |
| Creatinine (C) | 2.9 (2.6-3.2) | 2.6 (1.9-3.3) | 0.9 |
| Cysteine (D) | 0.0026 (0.0016-0.0036) | 0.0054 (0.0024-0.0084) | 2.1 |
| Ethanolamine (D) | 0.0075 (0.0054-0.0096) | 0.0060 (0.0040-0.0079) | 0.8 |
| ***Fructose (D)*** | ***0.13 (0.12-0.14)*** | ***0.43 (0.27-0.59)*** | ***3.4*** |
| Fructose bis-phosphate (D) | 0.097 (0.043-0.15) | 0.14 (0.067-0.22) | 1.5 |
| Fumaric acid (C) | 0.39 (0.36-0.41) | 0.36 (0.30-0.42) | 0.9 |
| GABA (C) | 5.3 (4.1-6.4) | 5.9 (3.3-8.4) | 1.1 |
| ***Glucose (D)*** | ***0.16 (0.025-0.29)*** | ***1.0 (0.67-1.4)*** | ***6.3*** |
| Glucose-6-phosphate (D) | 0.021 (0.0051-0.036) | 0.098 (-0.0029 to 0.20) | 4.7 |
| Glutamine (C) | 6. 6 (5.4-7.7) | 7.1 (5.1-9.1) | 1.1 |
| Glycerol (C) | 0.19 (0.17-0.21) | 0.19 (0.15-0.23) | 1.0 |
| Glycerol-3-phosphate (D) | 0.23 (0.13-0.34) | 0.36 (0.29-0.43) | 1.5 |
| Glycine (D) | 0.12 (0.09-0.14) | 0.11 (0.093-0.12) | 0.9 |
| Guanosine (D) | 1.01 (-1.1 to 3.1) | 0.75 (-0.26 to 1.8) | 0.7 |
| Hypoxanthine (D) | 2.2 (1.8-2.7) | 1.9 (1.4-2.33) | 0.8 |
| Inosine (D) | 0.79 (0.56-1.0) | 1.0 (0.80-1.3) | 1.3 |
| ***Iso-Erythritol (D)*** | ***0.00031 (0.00026-0.00037)*** | ***0.0012 (0.00089-0.0014)*** | ***3.7*** |
| ***Isoleucine (D)*** | ***0.10 (0.088-0.12)*** | ***0.17 (0.13-0.21)*** | ***1.7*** |
| L-Ascorbic acid (C) | 0.024 (0.0089-0.038) | 0.063 (0.026-0.10) | 2.7 |
| ***Lactic acid (C)*** | ***16.4 (15.4-17.3)*** | ***21.7 (19.7-23.7)*** | ***1.3*** |
| ***Leucine (D)*** | ***0.26 (0.22-0.29)*** | ***0.37 (0.31-0.43)*** | ***1.4*** |
| Malic acid (C) | 0.53 (0.47-0.6) | 0.56 (0.45-0.67) | 1.1 |
| ***Methionine (D)*** | ***0.053 (0.040-0.066)*** | ***0.031 (0.027-0.036)*** | ***0.6*** |
| Myo-Inositol phosphate (C) | 0.032 (0.024-0.040) | 0.044 (0.027-0.60) | 1.4 |
| Myo-Inositol (D) | 7.2 (6.3-8.2) | 7.8 (6.9-8.8) | 1.1 |
| N-acetylaspartic acid (D) | 9.4 (8.5-10.4) | 9.5 (8.2-10.7) | 1.0 |
| Palmitic Acid (D) | 2.8 (2.5-3.1) | 2.5 (2.2-2.9) | 0.9 |
| Pantothenic acid (D) | 0.039 (0.033-0.046) | 0.057 (0.039-0.078) | 1.5 |
| ***Phenylalanine (D)*** | ***0.36 (0.31-0.41)*** | ***0.19 (0.15-0.23)*** | ***0.5*** |
| Phosphoethanolamine (D) | 6.6 (5.4-7.9) | 6.1 (3.5-8.8) | 0.9 |
| Phosphoric acid (C) | 0.095 (0.070-0.12) | 0.11 (0.074-0.14) | 1.1 |
| Pyroglutamic acid (D) | 35.5 (32.3-38.8) | 39.4 (35.3-43.4) | 1.1 |
| Pyruvic Acid (D) | 0.012 (0.088-0.015) | 0.011 (0.0072-0.014) | 0.9 |
| ***Scyllo-inositol (D)*** | ***0.73 (0.60-0.86)*** | ***0.25 (0.20-0.31)*** | ***0.3*** |
| Serine (D) | 0.50 (0.34-0.66) | 0.57 (0.37-0.78) | 1.1 |
| ***Sorbitol (D)*** | ***0.20 (0.18-0.21)*** | ***0.82 (0.62-1.0)*** | ***4.2*** |
| Stearic Acid (D) | 1.2 (1.0-1.3) | 1.1 (0.93-1.3) | 1.0 |
| Succinic acid (C) | 0.041 (0.024-0.058) | 0.073 (0.037-0.11) | 1.8 |
| Sucrose (D) | 0.027 (0.019-0.036) | 0.84 (-1.02 to 2.69) | 30.6 |
| Tetradecanoic acid (C) | 0.043 (0.038-0.048) | 0.039 (0.033-0.045) | 0.9 |
| ***Threitol (D)*** | ***0.046 (0.043-0.050)*** | ***0.16 (0.13-0.19)*** | ***3.4*** |
| ***Threonine (D)*** | ***0.54 (0.49-0.59)*** | ***0.28 (0.25-0.32)*** | ***0.5*** |
| ***Tyrosine (D)*** | ***0.29 (0.24-0.34)*** | ***0.13 (0.10-0.17)*** | ***0.5*** |
| Uracil (C) | 0.13 (0.11-0.15) | 0.12 (0.10-0.14) | 0.9 |
| ***Urea (C)*** | ***22 (19-24.9)*** | ***31.8 (22.9-40.8)*** | ***1.4*** |
| Uridine (D) | 0.019 (0.010-0.027) | 0.037 (0.018-0.056) | 2.0 |
| Valine (D) | 0.31 (0.27-0.35) | 0.43 (0.31-0.54) | 1.4 |
| Control and diabetic values are mean (± 95% CI) of individual metabolite response-ratios to relevant internal standards. Abbreviations: (C) = confident, (D) = definitive identification. | | | |

| Suppl. Table 2 | | | |
| --- | --- | --- | --- |
| Concentrations of essential metals in whole-brain extracts from control and diabetic adult-male Wistar rats | | | |
| **Element** | **Control (n = 12)** | **Diabetic (n = 9)** | **Significance** |
| Na (mmol/wet-kg) | 40.5 (38.4 - 42.7) | 48.1 (37.3 - 58.9) | NS |
| Mg (mmol/wet-kg | 5.8 (5.3 - 6.3) | 5.9 (5.4 - 6.3) | NS |
| K (mmol/wet-kg) | 87.0 (81.3 - 92.7) | 90.7 (79.0 - 102.4) | NS |
| Ca (mmol/wet-kg) | 0.9 (0.8 - 1.1)^1^ | 1.0 (0.9 - 1.0) | NS |
| Mn (μmol/wet-kg) | 6.8 (6.2 - 7.3) | 7.3 (6.6 - 7.9) | NS |
| Fe (mmol/wet-kg) | 0.3 (0.3 - 0.4) | 0.3 (0.3 - 0.4) | NS |
| Cu (μmol/wet-kg) | 38.2 (32.6 - 43.8) | 43.7 (38.8 - 48.6) | NS |
| Zn (μmol/wet-kg) | 167.1 (144.8 - 189.4) | 187.7 (163.9 - 211.5) | NS |
| Se (μmol/wet-kg) | 2.0 (1.8 - 2.1) | 2.4 (2.2 - 2.6) | *** |
| Values are mean (±95% CI); ^1^, n = 11. Nonstandard abbreviations: NS, not significant; ***, P < 0.001, diabetic vs. control | | | |
