# Supplementary material for: Vitamin B5 (d-pantothenic acid) localizes in myelinated structures of the rat brain: Potential role for cerebral vitamin B5 stores in local myelin homeostasis
Source: Biochem Biophys Res Commun. 2020 Jan 29;522(1):220–5. doi: 10.1016/j.bbrc.2019.11.052 (PMC6977085; doi:10.1016/j.bbrc.2019.11.052)
Supplement: COI [file mmc2.zip › Ismail etal Main Manuscript_31-Oct-2019_v2_Final_corrected 10 NOV 2019_V2.docx]

**Vitamin B5 (D-pantothenic acid) localizes in myelinated structures of the rat brain: potential role for cerebral vitamin B5 stores in local myelin homeostasis**

Nashwah Ismail ^a^, Nina Kureishy ^a^, Stephanie J. Church ^a^, Melissa Scholefield ^a^, Richard D. Unwin ^a, b^, Jingshu Xu ^a^, Stefano Patassini ^a, c^, Garth J. S. Cooper ^a, d *^

^a^ *Centre for Advanced Discovery and Experimental Therapeutics, Division of Cardiovascular Sciences, School of Medical Sciences, Faculty of Biology, Medicine & Health, The University of Manchester, Manchester Academic Health Science Centre, Manchester M19 9NT, UK*

^b^ *Stoller Biomarker Discovery Centre and Division of Cancer Sciences, School of Medical Sciences, Faculty of Biology, Medicine and Health, The University of Manchester, CityLabs 1.0 (3rd Floor), Nelson Street*

^c^ *Owlstone Medical, Cambridge Science Park, Cambridge, CB4 0GJ, UK (present address)*

^d^ *School of Biological Sciences, Faculty of Science, University of Auckland, Private Bag 92 019, Auckland 1142, New Zealand*

*___________________________________________________________*

***** *Corresponding author*: Garth JS Cooper

*E-mail address*: garth.cooper@manchester.ac.uk

*Tel*: +44-161-275-1191

**Abstract**

Vitamin B5 (D-pantothenic acid; pantothenate) is an essential trace nutrient that functions as the obligate precursor of coenzyme A (CoA), through which it plays key roles in myriad biological processes, including many that regulate carbohydrate, lipid, protein, and nucleic acid metabolism. In the brain, acetyl-CoA is necessary for synthesis of the complex fatty-acyl chains of myelin, and of the neurotransmitter acetylcholine. We recently found that cerebral pantothenate is markedly lowered, averaging ~55% of control values in cases of Huntington’s disease (HD) including those who are pre-symptomatic, and that regions where pantothenate is lowered correspond to those which are more severely damaged. Here we sought to determine the previously unknown distribution of pantothenate in the normal-rat brain, and whether the diabetic rat might be useful as a model for altered cerebral pantothenate metabolism. We employed histological staining (Nissl) to identify brain structures; immunohistochemistry with anti-pantothenate antibodies to determine the distribution of pantothenate in caudate putamen and cerebellum; and gas-chromatography/mass-spectrometry to quantitate levels of pantothenate and other metabolites in normal- and diabetic-rat brain. Remarkably, cerebral pantothenate was almost entirely localized to myelin-containing structures in both experimental groups. Diabetes did not modify levels or disposition of cerebral pantothenate. These findings are consistent with physiological localization of pantothenate in myelinated white-matter structures, where it could serve to support myelin synthesis. Further investigation of cerebral pantothenate is warranted in neurodegenerative diseases such as HD and Alzheimer’s disease, where myelin loss is a known characteristic of pathogenesis.

***Key words****:*

Huntington’s disease; normal-rat brain; white matter; myelin synthesis; pantothenic acid; neurodegenerative disease

1. **Introduction**

Huntington's disease (HD) is a genetically-mediated neurodegenerative disorder where the etiological defect is a mutation in the huntingtin gene (*HTT*) that alters the structure of the huntingtin protein and initiates a cascade leading to dementia and premature death [1]. However, HD-evoked neurodegeneration is typically age-related, usually manifesting only in middle age, and the mechanisms that link the causative mutation to brain disease remain poorly understood. To further elucidate molecular links between *HTT* mutation and neurodegeneration in HD, and to identify novel therapeutic targets, we have performed case-control studies of post-mortem human brain by applying metabolomics [2]. We identified marked elevations in the polyol-pathway intermediates glucose, sorbitol, and fructose, and of urea in HD and also in Alzheimer’s disease (AD). Metabolic perturbations in affected brain regions of HD and AD closely resemble each other when viewed through the metabolomic lens [3], and their patterns also mimics those in diabetic nerve damage [4]. The signs of defective glucose utilization in affected brain regions found here are consistent with our findings in HD, AD, and diabetic neuropathy.

In untargeted and follow-up targeted case-control studies, we also identified pervasive lowering in HD brain of vitamin B5, the obligate precursor of CoA, which is essential for maintenance of normal intermediary metabolism [5]; notably, cerebral pantothenate deficiency was present even in cases of presymptomatic disease. CoA is an indispensable cofactor in all living organisms, where it functions as an acyl carrier and carbonyl-activating group in a number of central biochemical processes, including the tricarboxylic acid (TCA) cycle and fatty acid metabolism; it has been estimated that ~4% of all known human enzymes utilize CoA as an obligate cofactor [5, 6]. CoA plays additional, key specialized roles in the brain, where it mediates the synthesis of acetylcholine (ACh) [7], and of the complex fatty-acyl chains of myelin that are essential for myelin function [8], in particular the galactosylcerebrosides and galactosylsulphatides that underpin the insulating function of myelinated neurons, thus enabling saltatory neuronal conduction [8]. Furthermore, there is growing evidence that HD is characterized by myelin breakdown [9, 10], providing a further potential link between its pathogenesis and cerebral pantothenate deficiency.

Acetyl-CoA links glycolysis and pyruvate oxidation with the TCA cycle. In the presence of oxygen, acetyl-CoA transfers acetyl groups derived from glycolysis by the action of pyruvate dehydrogenase (PDH) into the TCA cycle [11]. We thus reasoned that pantothenate deficiency, leading to deficient CoA function, could provide a plausible mechanism to explain signs of defective glucose utilization in HD brain.

Here we aimed to localize pantothenate to structures in the brain of the rat, a model organism frequently used to study metabolic processes. There are substantive levels of pantothenate in all regions of normal human brain where measurements have been reported to date [2, 5], but to our knowledge there are no prior reports describing its localization to structures within gray or white matter. Furthermore, given the close similarities between patterns of metabolic disturbance in diabetic neuropathy and damaged brain regions in HD [2, 4], we analysed the cerebral disposition and levels of pantothenate, along with levels of other relevant metabolites in control and diabetic rats, employing a widely used model of diabetes, the streptozotocin (STZ)-diabetic rat; here, ‘pantothenate disposition’ means ‘the way in which pantothenate is located in relation to other tissue components’.

1. **Methods**
   1. *Animals*

Animal procedures were carried out under a UK Home Office project licence following approval by The University of Manchester’s Animal Welfare Ethics Review Committee, and this study is consistent with the Guide for the Care and Use of Laboratory Animals and the ARRIVE guidelines.

- 1. *Tissue processing and histological staining.*

Tissue was fixed, sectioned and stained with hematoxylin and eosin (H&E) (results not shown) and Nissl (Luxol Fast Blue/Cresyl Violet) stain, as detailed (Suppl. Methods).

- 1. *Immunohistochemistry*

Pantothenate was imaged by immunohistochemistry using a method based on an anti-pantothenate antiserum [12] and was otherwise performed as described (Suppl. Methods).

The specificity of the antibody we employed here for immunohistochemical detection of pantothenate in brain tissue has been comprehensively validated by the scientists who originally made it, and who have provided a detailed, comprehensive description of the specificity testing employed [12]. In brief, the antibodies have been assessed and characterized by ELISA tests [13]. After titration and for competition experiments, optimal dilution of the anti-pantothenate antiserum was observed at 1:60,000–1:70,000. This procedure was performed for each compound considered as a potential competitor, based in part on grounds of similarity of chemical structures to that of pantothenate. The specificity of the anti-pantothenate antibodies is very high, since the antiserum discriminates analogous structures very well: pyridoxine, choline, retinoic acid, alpha-tocopherol, vitamin C and D, and biotin; indeed, no cross-reactivity was observed with any of the competitor-molecules tested, which were examined as BSA conjugates [12]. The observed immunoreactivity in neural tissue disappears when the anti-pantothenate antiserum is pre-absorbed with an excess of pantothenate, and when the first and/or the second antibodies are omitted (as we re-verified in the current study) [12]. More detailed descriptions of the methods supporting the specificity of the anti-pantothenate antibody we employed here is presented in the following references [12, 13].

- 1. *Gas chromatography mass spectrometry (GC-MS)*

GC-MS was performed by using validated methods [2, 14] as otherwise detailed (Suppl. Methods).

- 1. *Derivatisation*

Metabolites were derivatised by validated methods as detailed (Suppl. Methods).

- 1. *Data processing for GC-MS*

We used ChromaTOF 4.5 (LECO) software for data processing as detailed (Suppl. Methods).

- 1. *Inductively-coupled plasma mass spectrometry (ICP-MS)*

50 ± 5 mg wet-weight samples were dissected using a metal-free ceramic scalpel and transferred into 2-ml microcentrifuge tubes (Eppendorf; Stevenage, UK) and pulse-centrifuged. Metals were measured using a validated method as described [5] and detailed (Suppl. Methods).

*2.8. Statistics*

Relative fold-changes were analysed in log_2_ space using Welch’s *t*-tests (modified for unequal variance) by using GraphPad v7.04 (Prism; La Jolla, CA), with adjustments for multiple comparisons through application of a 10% false discovery rate (FDR) correction [15].

*2.9. PCA and OPLS-DA*

Multivariate Principal Component Analysis (PCA) and Orthogonal Projections to Latent Structures Discriminant Analysis (OPLS-DA) were performed using R (MetaboAnalystR; version 1.0.1) to assess data quality and identify global metabolite differences in whole-brain extracts from control and diabetic rats. To assess the validity of the OPLS-DA, goodness-of-fit values of the model were calculated and then compared with the goodness-of-fit of 100 Y-permutated models. A Mann–Whitney U test (at a significance level of P < 0.05) was applied to test significance in the model. Multiple two-tailed *t*-tests were applied to determine whether relevant metabolite-abundance differences were observed, and to ascertain the statistical significance of our observations. We considered our entire datasets for multiple-comparisons analysis by applying a 10% FDR correction, and calculated fold-changes in metabolite abundances as the ratios of the means of the case-control group and have presented them here as diabetic/control (D/C) ratios (Suppl. Table 1, 2).

# Results and Discussion

This study was motivated by our recent observations using metabolomics that pantothenate is widely distributed in the normal human brain, and that its levels are widely depressed in cases of HD, including those with pre-symptomatic disease [5], implying that impaired pantothenate metabolism is an early event in the pathogenesis of neurodegeneration.

We aimed to improve understanding of the role of cerebral pantothenate deficiency in age-related neurodegeneration by determining its cerebral disposition in normal and diabetic rats [2, 16]. We performed complementary metabolomic and metallomic studies to characterise potential overlaps between cerebral metabolism in HD and diabetes, following our recent studies where we identified diabetes-like metabolic perturbations in HD-human brain [2].

We employed Nissl staining to visualise brain structures, and pantothenic-acid-selective immunohistochemistry to characterise its disposition in tissues from two regions, caudate putamen and cerebellum, in control and diabetic rats (Fig. 1). These regions were chosen because the former exemplifies a high-impact region in HD, whereas the latter represents a low-impact region [2, 16]. We verified staining specificity by suppression of the immunofluorescence signal upon omission of the primary antibody from the staining protocol (Fig. 1).

## Pantothenate immunoreactivity was widespread in caudate putamen and cerebellum (Fig. 1), consistent with results of our metabolomics studies in normal human brain [5], and metabolomic measurements made here in whole rat brain (Suppl. Table 1). The pattern of pantothenate distribution did not differ significantly between different non-diabetic rats, or between diabetic and control animals (Fig. 1).

## Pantothenate-staining intensity correlated closely with the distribution of myelinated structures as identified by Nissl staining in both brain regions (Fig. 1). To our knowledge, these observations concerning the localization of pantothenate in the brain are novel. Pantothenate localized specifically to the Nissl-positive structures in both regions.

## The presence of substantive pantothenate levels throughout the normal brain is consistent with the existence of pantothenate stores that could well participate in localized metabolic processes, such as those entailed in myelin synthesis. The localisation of pantothenate to myelinated structures suggests a functional relationship with important aspects of myelin structure and function. Consistently, acetyl-CoA and a number of acyl-CoAs are obligate precursors in myelin biosynthesis [8]; acetyl-CoA is also necessary for the production of ACh, a neurotransmitter that plays a central role in the maintenance of myelinated neurons [17].

We found that pantothenate disposition in myelinated structures is equivalent in caudate putamen and cerebellum from normal rats; nor did it differ between control and diabetic rats. We previously showed, by quantitative GC-MS metabolomics, that putamen and cerebellum from normal human brain contain large amounts of pantothenate, average concentrations being ~150μmol/wet-kg in cerebellum and ~60μmol/wet-kg in putamen [5].

The identification of pantothenate localized in myelin-containing structures provides new insights into potential mechanisms of age-related neurodegeneration in diseases such as HD, where demyelination is evident, loss of white matter is known to precede the onset of clinical symptoms in highly impacted regions [10, 18], and pantothenate is deficient [2, 5]. We suspect that these observations are linked, and hypothesize that deficient cerebral pantothenate stores could play a major role in the causation of demyelination in age-related neurodegenerative diseases.

These findings are consistent with the presence of large pantothenate stores in myelinated structures and imply the physiological need for a large, readily available pantothenate supply near its potential sites of utilization in the myelinated axonal structures of the white matter. These cerebral pantothenate stores, here described for the first time, are presumably necessary for the high rates of myelin synthesis required to maintain the integrity of myelinated neurons.

To our knowledge, our findings linking cerebral pantothenate deficiency neurodegeneration in HD are novel. We believe that cerebral pantothenate deficiency in HD is unlikely to be caused by insufficient dietary intake, since pantothenate is ubiquitous in food [19]; rather, it might be attributable to defective brain pantothenate uptake, perhaps through impaired function of its cognate transporter, SLC5A6 [5]; this hypothesis has yet to be tested experimentally. We interpret the magnitude of the lowered levels of cerebral pantothenate in HD as similar to those of other vitamins in other B-vitamin-deficiency diseases, where acquired deficiency of a specific vitamin can cause a cognate deficiency state leading to neurodegeneration and dementia (for example, beriberi caused by thiamine (vitamin B1) deficiency, or pernicious anaemia caused by vitamin B12 deficiency) [20]. We previously found that lowered cerebral pantothenate levels are prominent in regions subject to major HD-mediated damage such as the putamen, but occur also in areas commonly associated with minimal cell loss, including the cerebellum, superior frontal gyrus, cingulate gyrus, and hippocampus [5].

We also employed GC-MS metabolomics to compare brain-metabolite levels between control and diabetic rats in this case-control study, with data analysis by PCA and OPLS-DA (Fig. 2) [5]. This investigation was motivated by our prior studies of brain composition in HD and AD where we found several diabetes-like metabolic perturbations. Here we identified and quantitated the metabolites contained in whole-brain extracts, reproducibly measuring 56 metabolites in rat-brain tissue (Suppl. Table 1), of which 16 showed significant, consistent between-group differences by univariate analyses (Table 1). Vales for each metabolite are shown in Suppl. Table 1. These numerical data enable comparison with other metabolomic data sets. For example, they overlap with reported values from the brain in HD and AD cases [2, 3, 14], and in the peripheral nervous system in diabetic rats (sciatic nerve, dorsal root ganglion, and trigeminal ganglion) [4].

There was minimal overlap between control and diabetic metabolomes in the PCA (Fig. 2A) and OPLS-DA (Fig. 2B) plots: both provide strong evidence for distinct metabolic clustering between control and diabetic groups. Reproducibly altered metabolites included molecules from central metabolic pathways, including those with roles in glucose and amino-acid metabolism, alternative fuel sources, the TCA cycle, and urea metabolism. Cerebral levels of glucose, sorbitol, and fructose were all markedly increased in diabetic brain, consistent with elevations in diabetic neuropathy [4], and in brain regions in HD [2] and AD [3]. These findings are consistent with increased activity in the polyol pathway, and linked defects in flow through glycolysis and entry of acetyl groups from glycolysis into the TCA cycle catalysed by PDH. Consistently, PDH activity is reportedly diminished in AD- and HD-brain tissue [21]. These metabolic perturbations could be consistent with deficient pantothenate levels, since its obligate metabolite, CoA, is also expected to be deficient as a corollary of pantothenate deficiency. This hypothesis can now be tested by measuring pantothenate, CoA, acetyl-CoA, and acyl-CoA levels in relevant brain-tissue preparations.

As a result of impaired cerebral glucose utilisation in diabetes, the brain can shift towards using alternate fuel sources to meet its high energy needs, consistent with enhanced cerebral utilisation of ketone bodies and fatty acids [22]. Here we found levels of beta-hydroxybutyrate, glycerol-3-phosphate, lactate, threitol, iso-erythritol, scyllo-inositol, and xylitol to all be significantly higher in diabetic-rat brain. Lactic acid elevation is known to occur in diabetes, due to inadequate oxidative glucose utilization and elevated anaerobic respiration [23]; and beta-hydroxybutyric acid is a ketone body used for cerebral energy production that can partially compensate for lack of glucose utilisation, and whose production is increased in diabetes [24]. Some of these results are similar to findings in HD brain [25]. Several amino acids also displayed between-group differences: concentrations of threonine, phenylalanine, tyrosine, and methionine were decreased in diabetic-rat brain, whereas those of leucine, isoleucine, cysteine, alanine, valine, inosine, and pyroglutamic acid were elevated. However, pantothenate levels did not differ between cases and controls (Suppl. Table 1), so altered pantothenate metabolism did not contribute to the observed diabetic-brain phenotype.

Widespread copper deficiency is present in AD brain, but the effect of diabetes on cerebral metal levels has seldom been reported. Here, cerebral levels of the metalloid selenium were moderately higher in diabetic than control rats, potentially consistent with altered anti-oxidant defences, whereas levels of all essential metals, including copper, did not differ between diabetic and control animals.

## Our previous studies have established that pantothenate is distributed widely in the brain but there are few prior publications regarding its localization to specific brain structures. One report in the rat based on immunohistochemical methods suggested that it localises exclusively in one region, the lateral septal nucleus [12]. However, our recent metabolomic studies have shown that it is present in substantive quantities throughout the human brain [2, 5]. This distribution is consistent with its role as obligate precursor of CoA. Also, as CoA plays central roles throughout the brain, it makes sense that its obligate precursor, pantothenate, would have a matching distribution. It is possible that our current, optimized immunofluorescence method is more sensitive for detecting pantothenate than that used in the prior IHC study [12]. We show here that pantothenate is more concentrated in areas with higher content of myelinated structures. This may be due to the increased activity of these cells, which may therefore require increased CoA-dependent glucose metabolism to support TCA-cycle activity. PDH is central to the production of acetyl-CoA from CoA, and entry of the acetyl moiety into the TCA cycle. ACh is also implicated in the regulation of myelination but the precise roles by which ACh signaling contributes to this process remain poorly understood [17]. In part this is a consequence of the complexity of cholinergic signaling, which is mediated by a rich diversity of receptors and enzymes regulating ACh, compounded by the complexity of oligodendroglial development and myelination. Acetyl-CoA also serves as a critical factor for survival of cholinergic neurons.

## On the basis of this study, we conclude that the STZ-diabetic rat model is unsuitable for the study of pantothenate deficiency and its potential role in age-related dementia. There are possible reasons for this discrepancy. For example, a longer period (than 16 weeks) might be required for pantothenate deficiency to manifest. This study has shown that pantothenate was not perturbed in the diabetic-rat brain, whereas several of the other metabolic perturbations in diabetic-rat brain replicated those in cases of HD [2, 16, 26].

In conclusion, pantothenate localizes to myelinated structures in the white matter of the normal-rat brain, and this pattern is unchanged by diabetes. Locally-stored pantothenate may well be necessary to support the high rates of myelin synthesis required by the healthy brain [8]. Pantothenate localization was equivalent in both rat-brain regions studied: the caudate putamen and cerebellum. The lack of diabetes-evoked perturbations in cerebral pantothenate levels demonstrated here by immunofluorescence supports this metabolomic study, where pantothenate did not differ between control and diabetic tissue. Finally, this study supports the idea that pantothenate deficiency could play a significant role in the myelin loss, ACh deficiency, neurodegeneration, and cognitive impairment that occur in age-related dementias such as HD [10]. Further studies, especially of pantothenate treatment in at-risk patients are required to test this hypothesis.

**Conflict of interest**

None.

**Acknowledgements**

We thank Dr Michael Anderson, The University of Manchester and Cynthia Tse, the University of Auckland for management of the research, for proof-reading and submission of the manuscript. We also thank Peter Walker and Grace Bako of The University of Manchester Histology Facility for their assistance with the histology studies.

We acknowledge the following funding sources: the Endocore Research Trust (60187); the Oakley Mental Health Research Foundation NZ (3627092); the Medical Research Council (UK) Research Grant (MR/L010445/1); The University of Manchester; and the Northwest Regional Development Agency through a combined programme grant to CADET; and the Greater Manchester Comprehensive Local Research Network. The Histology Facility equipment was purchased with The University of Manchester Strategic Fund grants.

**References**

[1] R.G. Snell, J.C. MacMillan, J.P. Cheadle, I. Fenton, L.P. Lazarou, P. Davies, M.E. MacDonald, J.F. Gusella, P.S. Harper, D.J. Shaw, Relationships between trinucleotide repeat expansion and phenotypic variation in Huntington's disease, Nature Genet, 4 (1993) 303-397.

[2] S. Patassini, P. Begley, J. Xu, S.J. Church, S.J. Reid, E.H. Kim, M.A. Curtis, M. Dragunow, H.J. Waldvogel, R.G. Snell, R.D. Unwin, R.L.M. Faull, G.J.S. Cooper, Metabolite mapping reveals severe widespread perturbation of multiple metabolic processes in Huntington's disease human brain, Biochim Biophys Acta, 1862 (2016) 1650-1662.

[3] J. Xu, P. Begley, S.J. Church, S. Patassini, S. McHarg, N. Kureishy, K.A. Hollywood, H.J. Waldvogel, H. Liu, S. Zhang, W. Lin, K. Herholz, C. Turner, B.J. Synek, M.A. Curtis, J. Rivers-Auty, C.B. Lawrence, K.A.B. Kellett, N.M. Hooper, E.R.L.C. Vardy, D. Wu, R.D. Unwin, R.L.M. Faull, A.W. Dowsey, G.J.S. Cooper, Elevation of brain glucose and polyol-pathway intermediates with accompanying brain-copper deficiency in patients with Alzheimer’s disease: metabolic basis for dementia, Sci Rep, 6 (2016) 27524.

[4] O.J. Freeman, R.D. Unwin, A.W. Dowsey, P. Begley, S. Ali, K.A. Hollywood, N. Rustogi, R.S. Petersen, W.B. Dunn, G.J.S. Cooper, N.J. Gardiner, Metabolic dysfunction is restricted to the sciatic nerve in experimental diabetic neuropathy, Diabetes, 65 (2016) 228-238.

[5] S. Patassini, P. Begley, J. Xu, S.J. Church, S.J. Reid, H.J. Waldvogel, R.G. Snell, R.L.M. Faull, R.D. Unwin, G.J.S. Cooper, Cerebral vitamin B_5_ (D-pantothenic acid) deficiency as a potential cause of metabolic perturbation and neurodegeneration in Huntington’s disease, Metabolites, 9 (2019) 113.

[6] M. Daugherty, B. Polanuyer, M. Farrell, M. Scholle, A. Lykidis, V. de Crecy-Lagard, A. Osterman, Complete reconstitution of the human coenzyme A biosynthetic pathway via comparative genomics, J Biol Chem, 277 (2002) 21431–21439.

[7] A. Szutowicz, H. Bielarczyk, A. Jankowska-Kulawy, T. Pawelczyk, A. Ronowska, Acetyl-CoA the key factor for survival or death of cholinergic neurons in course of neurodegenerative diseases, Neurochem Res, 38 (2013) 1523-1542.

[8] S. Aggarwal, L. Yurlova, M. Simons, Central nervous system myelin: structure, synthesis and assembly, Trends Cell Biol, 21 (2011) 585-593.

[9] G. Bartzokis, P.H. Lu, T.A. Tishler, S.M. Fong, B. Oluwadara, J.P. Finn, D. Huang, Y. Bordelon, J. Mintz, S. Perlman, Myelin breakdown and iron changes in Huntington’s disease: pathogenesis and treatment implications, Neurochem Res, 32 (2007) 1655–1664.

[10] J. Bourbon-Teles, S. Bells, D.K. Jones, E. Coulthard, A. Rosser, A. Metzler-Baddeley, Myelin breakdown in human Huntington’s disease: multi-modal evidence from diffusion MRI and quantitative magnetization transfer, Neurosci, 403 (2019) 79-92.

[11] Z.H. Zhou, D.B. McCarthy, C.M. O'Connor, L.J. Reed, J.K. Stoops, The remarkable structural and functional organization of the eukaryotic pyruvate dehydrogenase complexes, Proc Natl Acad Sci USA, 98 (2001) 14802-14807.

[12] A. Mangas, J. Yajeya, N. Gonzalez, M. Husson, M. Geffard, R. Coveñas, Detection of pantothenic acid-immunoreactive neurons in the rat lateral septal nucleus by a newly developed antibody, Folia Histochem Cytobiol, 54 (2016) 186–192.

[13] A. Mangas, R. Coveñas, D. Bodet, M. Geffard, L.A. Aguilar, J. Yajeya, Immunocytochemical visualization of D-glutamate in the rat brain, Neurosci, 144 (2007) 654–664.

[14] J. Xu, P. Begley, S.J. Church, S. Patassini, K.A. Hollywood, M. Jüllig, M.A. Curtis, H.J. Waldvogel, R.L.M. Faull, R.D. Unwin, G.J.S. Cooper, Graded perturbations of metabolism in multiple regions of human brain in Alzheimer's disease: Snapshot of a pervasive metabolic disorder, Biochim Biophys Acta, 1862 (2016) 1084-1092.

[15] Y. Benjamini, Y. Hochberg, On the adaptive control of the false discovery rate in multiple testing with independent statitstics, J Educ Behav Stat, 25 (2000) 60-83.

[16] S. Patassini, P. Begley, S.J. Reid, J. Xu, S.J. Church, M. Curtis, M. Dragunow, H.J. Waldvogel, R.D. Unwin, R.G. Snell, R.L.M. Faull, G.J.S. Cooper, Identification of elevated urea as a severe, ubiquitous metabolic defect in the brain of patients with Huntington's disease, Biochem Biophys Res Commun, 468 (2015) 161-166.

[17] R.D. Fields, D.J. Dutta, J. Belgrad, M. Robnett, Cholinergic signaling in myelination, Glia, 65 (2017) 687–698.

[18] A. Ciarmiello, M. Cannella, S. Lastoria, M. Simonelli, L. Frati, D.C. Rubinsztein, F. Squitieri, Brain white-matter volume loss and glucose hypometabolism precede the clinical symptoms of Huntington's disease, J Nucl Med, 47 (2006) 215-222.

[19] R.B. Rucker, K. Bauerly, Pantothenic acid, in: J. Zempleni, J.W. Suttie, J.F. Gregory III, P.J. Stover (Eds.) Handbook of Vitamins, Fifth Edition, CRC Press, Boca Raton, FL, USA, 2014, pp. 325-349.

[20] J. Zempleni, J.W. Suttie, J.F. Gregory III, P.J. Stover (Eds.), Handbook of Vitamins, Fifth ed., CRC Press, Boca Raton, FL, 2014.

[21] S. Sorbi, E.D. Bird, J.P. Blass, Decreased pyruvate dehydrogenase complex activity in Huntington and Alzheimer brain, Ann Neurol, 13 (1983) 72-78.

[22] J. Wahren, K. Ekberg, E. Fernqvist-Forbes, S. Nair, Brain substrate utilisation during acute hypoglycaemia, Diabetologia, 42 (1999) 812–818.

[23] K. Kasischke, Lactate fuels the neonatal brain, Front Neuroenergetics, 3 (2011) 1–3.

[24] L. Laffel, Ketone bodies: a review of physiology, pathophysiology and application of monitoring to diabetes, Diabetes Metab Res Rev, 15 (1999) 412–426.

[25] W.R.W. Martin, M. Wieler, C.C. Hanstock, Is brain lactate increased in Huntington’s disease?, J Neurol Sci, 263 (2007) 70–74.

[26] R.R. Handley, S.J. Reid, R. Brauning, P. Maclean, E.R. Mears, I. Fourie, S. Patassini, G.J.S. Cooper, S.R. Rudiger, C.J. McLaughlan, P. Verma, J.F. Gusella, M.E. MacDonald, H.J. Waldvogel, C.S. Bawden, R.L.M. Faull, R.G. Snell, Brain urea increase is an early Huntington’s disease pathogenic event observed in a prodromal transgenic sheep model and HD cases, Proc Natl Acad Sci USA, 114 (2017) E11293-E11302.

**Figure legends**

500µm

100µm

**Fig. 1.** Localization of pantothenic acid to myelinated structures in the caudate putamen (A-H) and cerebellum (I-P) of control and diabetic adult-male Wistar rats. Nissl-stained sagittal sections of representative whole rat brains where *open* *black* boxes indicate sites whence serial sections were taken (A, E, I, M). Serial sections of caudate putamen were from control (B-D) and diabetic (F-H) rats; and corresponding serial cerebellar sections from control (J-L) and diabetic (N-P) rats; studies are representative of N = 5 control and N = 6 diabetic animals. Serial sections were visualized by Nissl staining (Luxol Fast Blue/Crystal Violet), where myelin-containing structures are *dark blue* (*black arrows*) and *punctate* neuropil (representing rough endoplasmic reticulum) is *purple* (B, F, J, N). Immunofluorescent staining employed DAPI for cell nuclei (*blue*) and a primary antibody raised against conjugated D-pantothenic acid with Alexafluor 568-labelled secondary antibody, where pantothenic acid-containing structures are *red* (*white arrows*) (C, G, K, O), illustrating localization mainly to myelinated structures in the corresponding serial Nissl-stained sections. *Bottom row* shows immunofluorescence-stained sections where the primary antibody was omitted (D, H, L, P).

**Fig. 2**. Two-dimensional (A) PCA and (B) OPLS-DA plots of metabolites determined in control or diabetic rat whole-brain tissue extracts analysed by GC-MS metabolomics. (A) The PCA plot demonstrates essentially complete class separation between metabolites from diabetic (N = 9, blue circles) and control (N = 10, red circles) extracts, where the first principle component (PC1) explains 26.8% of the total variance, and the second (PC2) a further 14.2%; the compact clustering of the QC replicates (green circles) indicates the high reproducibility of the GC-MS analysis. (B) OPLS-DA model of diabetic (blue circles) and control (red circles) cases confirming definite class separation between the two groups, where the T-score was 23.9% and the orthogonal T-score was 18.3%. The PCA results support the reliability of the OPLS-DA model. The coloured regions around each group represent 95% confidence ellipsoids. Nonstandard abbreviations: C, control; D, diabetic; QC, quality control).

| Table 1  Metabolites whose abundance was significantly altered in diabetic rat-brain tissue. Fold-changes are diabetic/control. | | | | |
| --- | --- | --- | --- | --- |
| **Metabolite** | **Control Mean** | **STZ Mean** | **Fold Change** | **Significance** |
| ***Glucose and related metabolites*** | |  |  |  |
| Glucose | 0.16 (0.025-0.29) | 1.0 (0.66-1.4) | 6.3 | *** |
| Fructose | 0.13 (0.12-0.14) | 0.43 (0.27-0.59) | 3.4 | *** |
| Sorbitol | 0.20 (0.18-0.21) | 0.82 (0.62-1.0) | 4.2 | *** |
| ***Alternative Fuel Sources*** |  |  |  |  |
| beta-Hydroxybutyric acid | 0.0024 (0.0017-0.0030) | 0.0086 (0.0041-0.013) | 3.6 | ** |
| Lactic Acid | 16.4 (15.4-17.3) | 21.7 (19.7-23.7) | 1.3 | *** |
| Threitol | 0.046 (0.043-0.050) | 0.16 (0.13-0.19) | 3.4 | *** |
| Iso-Erythritol | 0.00031 (0.00026-0.00037) | 0.0012 (0.00089-0.0014) | 3.7 | *** |
| Scyllo-Inositol | 0.73 (0.60-0.86) | 0.25 (0.20-0.30) | 0.3 | *** |
| ***TCA and Urea Cycles*** |  |  |  |  |
| Urea | 22 (19-24.9) | 31.8 (22.9-40.8) | 1.4 | * |
| 2-Oxoglutaric acid | 0.12 (0.088-0.15) | 0.22 (0.17-0.28) | 1.9 | *** |
| ***Amino Acids*** |  |  |  |  |
| Threonine | 0.54 (0.49-0.59) | 0.28 (0.27-0.32) | 0.5 | *** |
| Phenylalanine | 0.36 (0.31-0.41) | 0.19 (0.15-0.23) | 0.5 | *** |
| Tyrosine | 0.29 (0.24-0.34) | 0.13 (0.10-0.17) | 0.5 | *** |
| Isoleucine | 0.10 (0.088-0.12) | 0.17 (0.13-0.21) | 1.7 | ** |
| Leucine | 0.26 (0.22-0.29) | 0.37 (0.31-0.49) | 1.4 | ** |
| Methionine | 0.053 (0.040-0.066) | 0.031 (0.027-0.036) | 0.6 | ** |
| Data are ratios of responses to appropriate internal standards. Changes with P < 0.05 (10% FDR) are considered significant. Abbreviations: *, P < 0.05; **, P < 0.01; ***, P < 0.001, diabetic vs control. | | | | |

**Fig. 1**


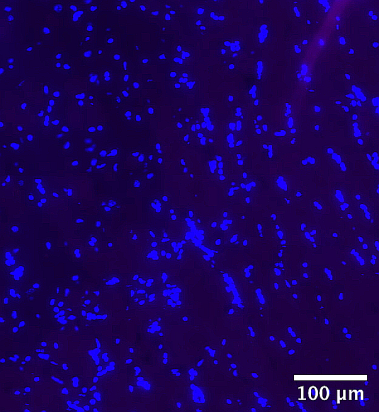

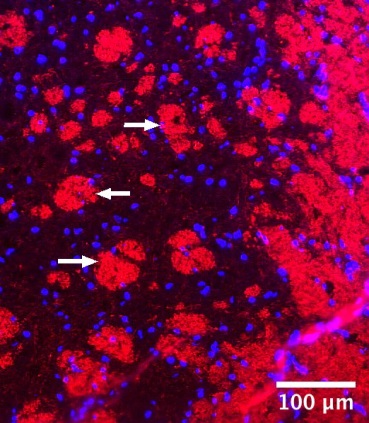

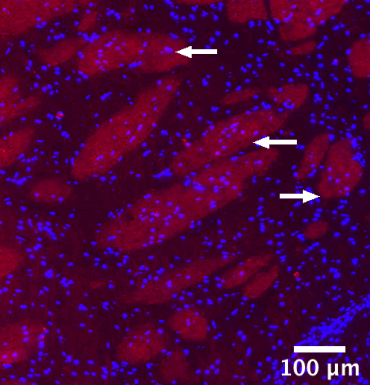

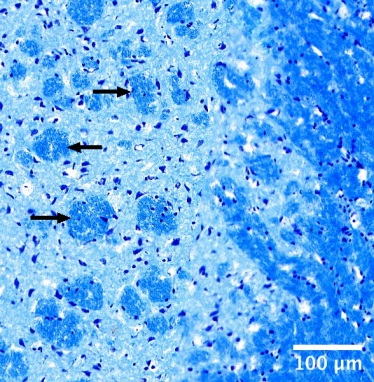

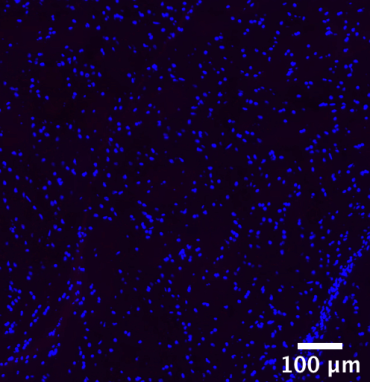

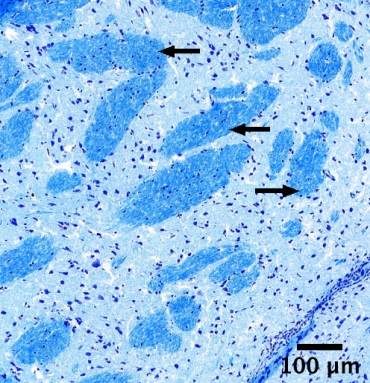


**A**

**B**

**E**

**F**

**C**

**G**

**H**

**D**


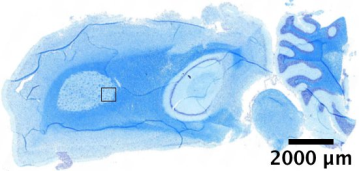

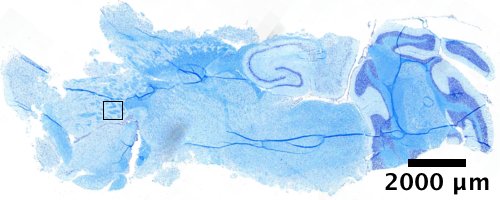

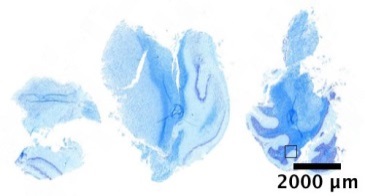

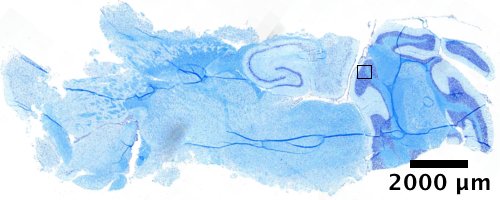


**I**

**M**


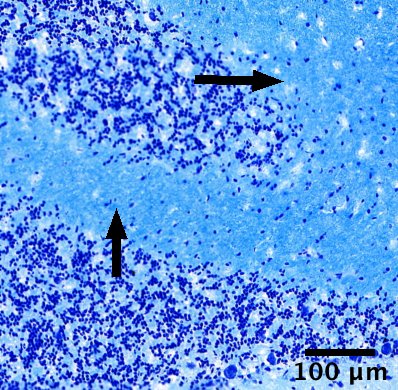


**J**


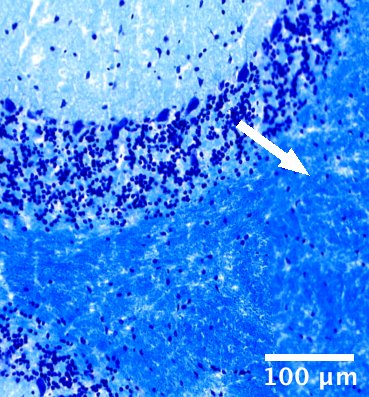


**N**


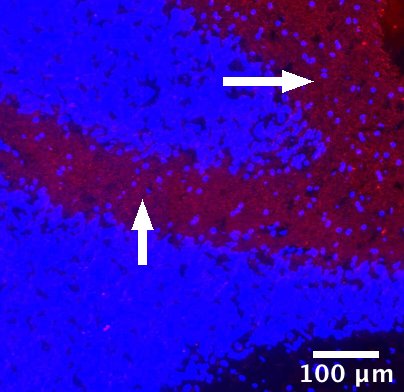


**K**


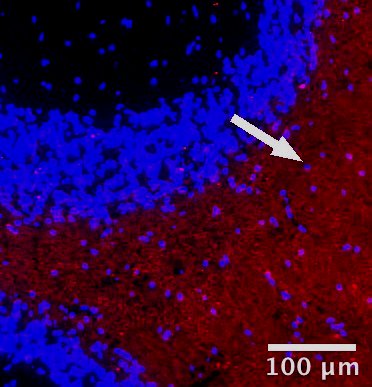


**O**


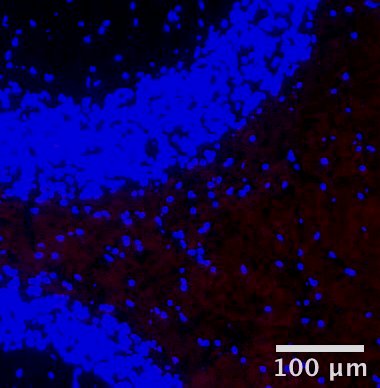

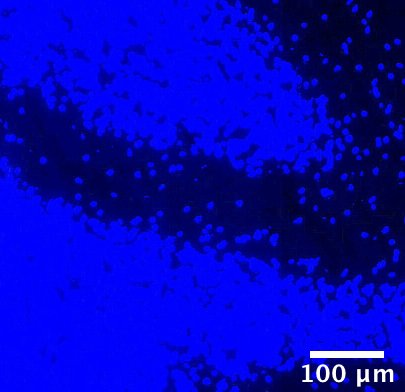


**L**

**P**
