# Supplementary material for: Vitamin B5 (d-pantothenic acid) localizes in myelinated structures of the rat brain: Potential role for cerebral vitamin B5 stores in local myelin homeostasis
Source: Biochem Biophys Res Commun. 2020 Jan 29;522(1):220–5. doi: 10.1016/j.bbrc.2019.11.052 (PMC6977085; doi:10.1016/j.bbrc.2019.11.052)
Supplement: COI [file mmc2.zip › Highlights Ismail et al_2019.docx]

**Ismail et al submission**

**Highlights**

- Vitamin B5 (pantothenate) is the obligate precursor of acetyl-coenzyme A (acetyl-CoA)
- Acetyl-CoA is essential for the cerebral synthesis of acetylcholine and myelin
- We determined the cerebral disposition of pantothenate in normal and diabetic rats
- Pantothenate was mainly localized to myelin-containing structures in both groups
- Localization of pantothenate to white-matter structures could support cerebral myelin synthesis
