# Supplementary figures and images for: Vitamin B5 (d-pantothenic acid) localizes in myelinated structures of the rat brain: Potential role for cerebral vitamin B5 stores in local myelin homeostasis
Source: Biochem Biophys Res Commun. 2020 Jan 29;522(1):220–5. doi: 10.1016/j.bbrc.2019.11.052 (PMC6977085; doi:10.1016/j.bbrc.2019.11.052)

**Fig. 2**

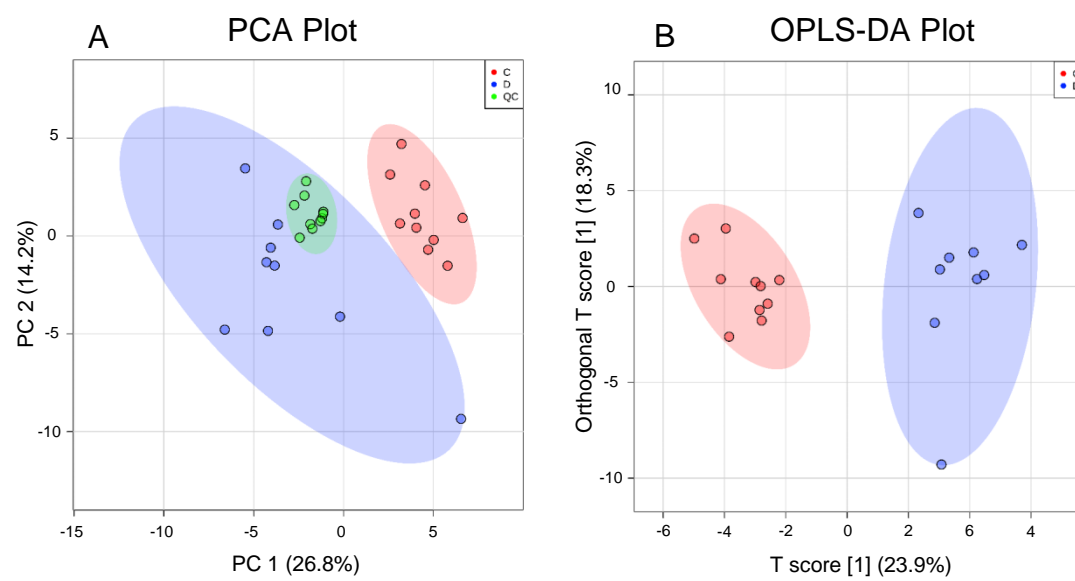

Supplement: COI [file mmc2.zip › Ismail etal MS fig 2.pdf]
